# Plasma Fibrinogen-to-Fractional Exhaled Nitric Oxide Ratio (FFR) as an Emerging Biomarker in Bronchiectasis

**DOI:** 10.3390/jcm15093383

**Published:** 2026-04-28

**Authors:** Andreas M. Matthaiou, Nikoleta Bizymi, Ioannis Tomos, Konstantina Symvoulaki, Christos Skiadas, Georgios Pitsidianakis, Adamantia Liapikou, Nikolaos Tzanakis, Katerina M. Antoniou

**Affiliations:** 1 Laboratory of Molecular and Cellular Pneumonology, School of Medicine, University of Crete, 70013 Heraklion, Greece; matthaiou.andreas@gmail.com (A.M.M.); nikoletabizymi@yahoo.gr (N.B.); 2 Department of Respiratory Medicine, University Hospital of Heraklion, 71500 Heraklion, Greece; 3 5th Department of Respiratory Medicine, Sotiria Thoracic Diseases General Hospital of Athens, 11527 Athens, Greece; 4 Respiratory Physiology Laboratory, Medical School, University of Cyprus, Nicosia 2029, Cyprus; 5 Department of Nursing, School of Health Sciences, National and Kapodistrian University of Athens, 11527 Athens, Greece; 6 School of Medicine, European University Cyprus, Nicosia 2404, Cyprus; 7 Department of Medical Imaging, University Hospital of Heraklion, 71500 Heraklion, Greece

**Keywords:** airway inflammation, biomarker, bronchiectasis, disease activity, disease severity, fibrinogen, fractional exhaled nitric oxide

## Abstract

**Background and Aims**: Plasma fibrinogen and fractional exhaled nitric oxide (FeNO) reflect neutrophilic and eosinophilic airway inflammation, respectively, and are associated with disease activity and severity in different directions in bronchiectasis. This study aimed to concurrently investigate fibrinogen and FeNO and further evaluate the clinical importance of fibrinogen-to-FeNO ratio (FFR) as a composite biomarker in bronchiectasis. **Methods**: This was a two-centre, observational, cross-sectional study involving stable bronchiectasis patients. Fibrinogen, FeNO, and the ratio of their normalised values (FFR) were investigated in relation to clinical indicators of disease activity and severity, including respiratory symptoms, inflammatory markers, pulmonary function, radiological extent, airway infection, severity scores, and patient-reported outcomes. **Results**: FFR was correlated with both circulating neutrophils (*r* = 0.36, *p* = 0.04) and eosinophils (*r* = −0.39, *p* = 0.03) and, more strongly compared to fibrinogen and FeNO, with the percentage of predicted forced expiratory volume in the 1st second (*r* = −0.61, *p* < 0.001). Interestingly, only FFR was found to be higher in patients with *Pseudomonas aeruginosa* isolation in respiratory secretions (*p* < 0.01). In receiver operating characteristic curves, FFR showed good discriminatory ability to differentiate patients with any level (AUC: 0.80, 95% CI: 0.64–0.96) or a severe level (AUC: 0.83, 95% CI: 0.64–1.00) of pulmonary functional impairment and patients with severe disease (AUC: 0.78, 95% CI: 0.62–0.94). **Conclusions**: FFR emerges as a candidate biomarker capturing the balance between neutrophilic and eosinophilic inflammation and the net disease activity and severity in bronchiectasis.

## 1. Introduction

Bronchiectasis is a chronic airway disease involving the permanent dilatation of the bronchial tree as a result of a wide spectrum of underlying conditions triggering a pathogenetic vicious vortex of airway structural distortion, mucociliary clearance impairment, chronic infection, and immune dysregulation [1,2]. Neutrophilic inflammation of the airways has traditionally been considered as a hallmark of bronchiectasis, although recent studies have highlighted eosinophilic inflammation in up to approximately one-third of patients in the absence of asthma or allergic bronchopulmonary aspergillosis [3,4]. Importantly, both eosinophilia and eosinopenia are followed by increased severity of the disease [5], while systemic inflammation is associated with a greater risk of severe exacerbations [6]. Chronic colonisation of both the upper and lower airway is common among bronchiectasis patients, with *Pseudomonas aeruginosa* (*P. aeruginosa*) as the cardinal pathogen associated with worse disease outcomes [7,8].

Beyond its central role in the coagulation cascade, fibrinogen also demonstrates multifaceted functions in inflammation as a systemic acute-phase protein by its interaction with neutrophils and other immune cells, promoting their transmigration into injured tissues and enhancing their involvement in inflammatory pathways [9,10]. Among the diverse molecular biomarkers of inflammation in bronchiectasis, fibrinogen appears to most consistently reflect disease severity, as it positively correlates with validated severity scores, functional status deterioration, pulmonary functional impairment, and chronic airway infection by *P. aeruginosa* [11,12,13,14,15,16].

Although the local production of nitric oxide (NO) by the airway epithelium, as expressed by the fractional exhaled NO (FeNO), has traditionally been considered as a biomarker of type 2-high inflammation in asthma [17] and associated bronchiectasis [18], growing evidence has indicated its clinical value in non-asthmatic bronchiectasis. Lower FeNO levels are generally observed in stable bronchiectasis compared to asthma and chronic obstructive pulmonary disease [19,20] and are associated with unfavourable clinical features [21,22]. FeNO is strongly linked to type 2-high inflammation in bronchiectasis, even in the absence of asthma, and is correlated with circulating eosinophils and serum immunoglobulin E [3,23].

The concepts of disease activity and severity have been recently distinguished in bronchiectasis. Disease activity denotes the ongoing inflammatory and infectious burden, whereas disease severity represents the cumulative structural and functional damage of the lungs [24]. Previous observations from our group pointed out the increasing trend of plasma fibrinogen and a decreasing trend of FeNO with worsening pulmonary function in bronchiectasis [25,26]. We also demonstrated that both biomarkers correlate with neutrophilic and eosinophilic inflammation, respectively [26].

The associations of multiple aspects of disease activity and severity with both FeNO, as a local marker of eosinophilic inflammation, and fibrinogen, as a systemic marker of neutrophilic inflammation, have been extensively delineated in bronchiectasis. However, to the best of our knowledge, their comparative alterations and clinical importance as pairwise inverse biomarkers, in terms of their site of origin, type of inflammation they are involved in, and favourability of their associated clinical features, have not been investigated. Our aim was to simultaneously assess the two biomarkers to further derive a composite inflammatory ratio, i.e., plasma fibrinogen-to-FeNO ratio (FFR), and elucidate its capacity to indicate the net inflammatory polarity and the net activity and severity in bronchiectasis.

## 2. Materials and Methods

### 2.1. Study Design and Participants

This was a two-centre, observational, cross-sectional study investigating the clinical importance of fibrinogen, FeNO, and FFR in bronchiectasis. Participants included adult stable bronchiectasis patients recruited from two large referral centres in Greece, the Sotiria Thoracic Diseases General Hospital of Athens and the University General Hospital of Heraklion, from July 2022 to July 2025. All patients were recruited during their regular follow-up in outpatient settings at the two hospitals. Eligibility criteria included a confirmed diagnosis of bronchiectasis based on the findings of chest high-resolution computed tomography in accordance with the 2019 British Thoracic Society guidelines [27]. Exclusion criteria were age > 85 years, presence of malignant, haematological, autoimmune, or other chronic infectious comorbidities, history of organ or tissue transplantation, and acute exacerbation or any other acute infection within the previous four weeks, to avoid biased results potentially attributed to other acute or chronic disease states or inflammaging.

### 2.2. Clinical Workup

Clinical workup at the recruitment and enrolment of eligible participants included, in this order: (i) clinical data collection from medical records; (ii) completion of the Quality of Life–Bronchiectasis (QOL-B) patient-reported outcome measure [28]; (iii) FeNO measurement in accordance with international technical standards [29]; (iv) spirometry in accordance with the international technical standards for the measurement of the forced expiratory volume in the 1st second (FEV_1_), the forced vital capacity (FVC), and the FEV_1_/FVC ratio, all expressed as absolute values and percentages of their predicted values (%Pred) [30]; and (v) laboratory testing for complete blood count, plasma fibrinogen, erythrocyte sedimentation rate, and serum C-reactive protein (CRP). The most recent high-resolution computed tomography scans were further re-interpreted by a radiology specialist (C.S.) with experience in chest imaging, and the Bronchiectasis Radiologically Indexed Computed Tomography Score (BRICS) was calculated [31]. The validated severity scores for the Bronchiectasis Severity Index (BSI) and FEV_1_–Age–Colonisation by *P. aeruginosa*–Radiological Extent–Dyspnoea (FACED) were additionally calculated [32,33].

### 2.3. Plasma Fibrinogen Measurement

Plasma fibrinogen was measured using the Clauss method [34]. In brief, blood was collected in a sodium citrate-containing tube and centrifuged for the separation of blood components and the isolation of platelet-poor plasma from the cellular elements. Plasma was then pre-diluted to minimise interference by anticoagulants or fibrin degradation products. A pre-warmed thrombin reagent was added to the diluted plasma after its incubation at 37 °C. The clotting time, which is inversely proportional to the fibrinogen concentration, was measured in seconds from the exact moment of the addition of the thrombin reagent. The fibrinogen concentration was finally calculated by the interpretation of the clotting time using a standard curve.

### 2.4. FeNO Measurement

FeNO measurement was performed using NIOX VERO^®^ (NIOX Group PLC, Oxford, United Kingdom) following international technical standards [29]. The patient inhaled to total lung capacity through the device’s filter and then exhaled at a constant flow rate (50 mL/s) for at least six seconds, maintaining a steady pressure to keep the soft palate closed and prevent nasal nitric oxide contamination. The device continuously analysed the exhaled air and reported the FeNO value in ppb. FeNO measurement preceded spirometry in all patients.

### 2.5. Calculation of FFR

For the calculation of FFR, prior to analysis, fibrinogen and FeNO values were normalised to their population-based upper limits of normal. This was done for two reasons. First, the two variables were measured in different units, i.e., fibrinogen in mg/dL and FeNO in ppb. Second, the two variables were measured in different scales, meaning that the same absolute change could imply very different magnitudes of biopathological changes expressed by the two variables. The upper limit of normal for fibrinogen was set at 400 mg/dL, consistent with widely accepted reference ranges, whereas the upper limit of normal for FeNO was defined as 25 ppb, in accordance with previously published data [3,21,22]. The ratio of the normalised fibrinogen value to the normalised FeNO value was subsequently calculated and used as FFR for all analyses.

### 2.6. Data Processing and Statistical Analysis

Categorical variables were expressed as frequencies and percentages. Continuous variables were tested for normality using the Shapiro–Wilk test and were presented as mean ± standard deviation for normally distributed data or median (interquartile range) for non-normal distributions. Parametric and non-parametric statistical tests were appropriately conducted using the GraphPad Prism version 10.0.0 for Windows (GraphPad Software, Boston, MA, USA). Comparisons between two or multiple groups were investigated using the parametric *t*-test or one-way analysis of variance, respectively, for normally distributed data or using the non-parametric Mann–Whitney U test or Kruskal–Wallis test, respectively, for non-normally distributed data. For the identification of clinically important aspects of plasma fibrinogen, FeNO, and plasma fibrinogen-to-FeNO ratio (FFR), exploratory data analysis was conducted to investigate their correlations with a wide range of clinical and laboratory features and patient-reported outcomes. Multiple comparisons correction was not implemented owing to the explorative nature of the analysis. The parametric Pearson’s correlation coefficient and the non-parametric Spearman’s rank correlation coefficient were used, as appropriate, for the identification of correlations between different parameters. Analysis of contingency tables was performed using Fischer’s exact test. Receiver operating characteristic curve analyses were conducted to assess the discriminative ability of FFR and its components for identifying patients with severe bronchiectasis, defined according to the Bronchiectasis Severity Index (≥9). The area under the curve (AUC) with 95% confidence intervals (CIs) was calculated to quantify diagnostic performance. The acceptable level of statistical significance was set at *p* < 0.05 and the trend at 0.05 ≤ *p* < 0.10. The study included 48 patients, providing 80% power at α = 0.05 to detect correlations with *r* ≥ 0.40.

## 3. Results

### 3.1. Study Participants and Clinical Features

A total of 48 stable bronchiectasis patients were included in this study. Mean fibrinogen was found at 399.9 ± 95 mg/dL, with 19 (42.2%) patients presenting hyperfibrinogenaemia (more than 400 mg/dL). Median FeNO was found at 18 (15.5) ppb, with 9 (25%) patients presenting values between 25 and 49 ppb and two (5.6%) patients at 50 ppb or more. Median FFR was measured in 33 patients and was found at 1.2 (1.3). All patients’ clinical and laboratory features and patient-reported outcomes are summarised in Table 1.

### 3.2. Differences in Fibrinogen, FeNO, and FFR Across Bronchiectasis Patient Groups Stratified by Clinical Indicators of Disease Severity

**Figure 1:**
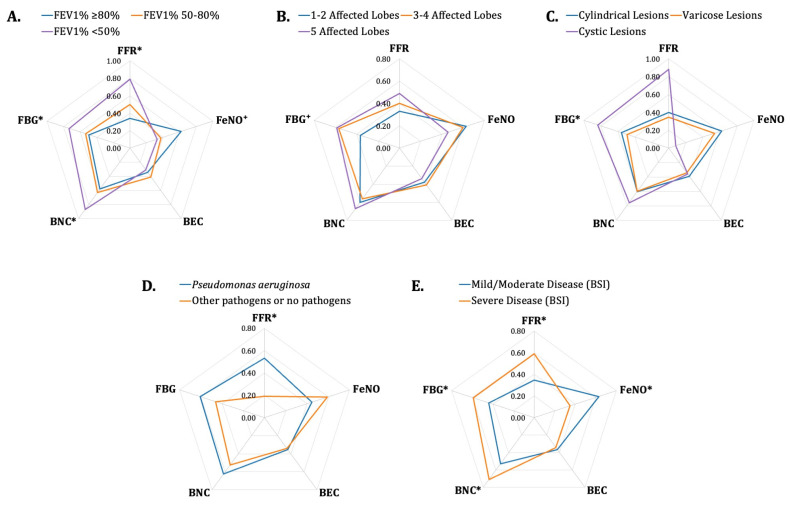
Comparison of fibrinogen, FeNO, FFR, BNC, and BEC across bronchiectasis patient groups, stratified by clinical indicators of disease severity. The radar plots compare the medians of fibrinogen, FeNO, FFR, BNC, and BEC across bronchiectasis patient groups, stratified by selected clinical indicators of disease severity, particularly pulmonary function, radiological extent, airway pathogen isolation, and validated severity scores. The corners of the hexagons represent the medians of the normalised values of the common logarithms of the variables in a scale from zero to one. Comparisons were conducted using the Mann–Whitney U test or the Kruskal–Wallis test, as appropriate, for non-normally distributed variables and the *t*-test or the one-way analysis of variance, as appropriate, for normally distributed variables. Statistically significant differences (*p* < 0.05) are indicated by an asterisk (*), while trends (0.05 ≤ *p* < 0.10) are indicated by a dagger (^†^). Panel (**A**): Patients with severe pulmonary functional impairment, i.e., FEV_1_%Pred < 50%, demonstrated significantly higher values of FFR (*p* = 0.01), fibrinogen (*p* = 0.03), and BNC (*p* = 0.02), and tended to demonstrate lower values of FeNO (*p* = 0.06) compared to patients with mild or moderate impairment. Panel (**B**): Patients with more extended disease, i.e., in five lung lobes, tended to demonstrate higher values of fibrinogen (*p* = 0.05) compared to patients with more localised disease, i.e., in one or two lung lobes. Panel (**C**): Patients with cystic bronchiectasis demonstrated significantly higher values of fibrinogen (*p* = 0.03) compared to patients with cylindrical or varicose bronchiectasis. Panel (**D**): Patients with isolation of *P. aeruginosa* in respiratory secretions (with or without isolation of other pathogens) demonstrated significantly higher values of FFR (*p* < 0.01) compared to patients with isolation of other pathogens only or with unremarkable respiratory secretion cultures. Outliers, defined by an absolute modified z-score > 3.5, were excluded from the analysis. Panel (**E**): Patients with severe disease according to BSI demonstrated significantly higher values of FFR (*p* < 0.01), fibrinogen (*p* < 0.01), and BNC (*p* < 0.001) and significantly lower values of FeNO (*p* = 0.01) compared to patients with mild or moderate disease. BEC: blood eosinophil count; BNC: blood neutrophil count; BSI: Bronchiectasis Severity Index; FBG: plasma fibrinogen; FeNO: fractional exhaled nitric oxide; FEV_1_%Pred: percentage of predicted value of forced expiratory volume in the 1st second; FFR: plasma fibrinogen-to-fractional exhaled nitric oxide ratio; *P. aeruginosa*: *Pseudomonas aeruginosa*.

We compared fibrinogen, FeNO, and FFR across patient groups stratified by selected clinical indicators of disease severity using the Mann–Whitney U test or the Kruskal–Wallis test, as appropriate. The results of the three variables, along with blood neutrophil and eosinophil counts, are comparatively illustrated in radar plots in Figure 1. Patients with severe pulmonary functional impairment, i.e., FEV_1_%Pred < 50%, demonstrated significantly higher values of FFR (*p* = 0.01) and fibrinogen (*p* = 0.03) and tended to demonstrate lower values of FeNO (*p* = 0.06) compared to patients with mild or moderate impairment (Figure 1A). Regarding radiological extent, patients with cystic or diffuse bronchiectasis demonstrated higher values of fibrinogen (*p* = 0.05 and *p* = 0.03, respectively) compared to patients with cylindrical or varicose bronchiectasis or more localised disease (Figure 1B,C). Interestingly, only FFR, but not fibrinogen or FeNO independently, differed significantly in patients with isolation of *P. aeruginosa* in respiratory secretions (*p* < 0.01), with higher values in the presence of the cardinal pathogen (Table 2 and Figure 1D), suggesting systemic inflammatory dominance rather than direct pathogen effect. Finally, patients with severe disease according to BSI presented significantly higher values of FFR (*p* = 0.01) and fibrinogen (*p* = 0.01) and significantly lower values of FeNO (*p* = 0.01) compared to patients with mild or moderate disease (Figure 1E).

### 3.3. Correlations of Fibrinogen, FeNO, and FFR with Clinical Indicators of Disease Activity and Severity and Patient-Reported Outcomes

**Figure 2:**
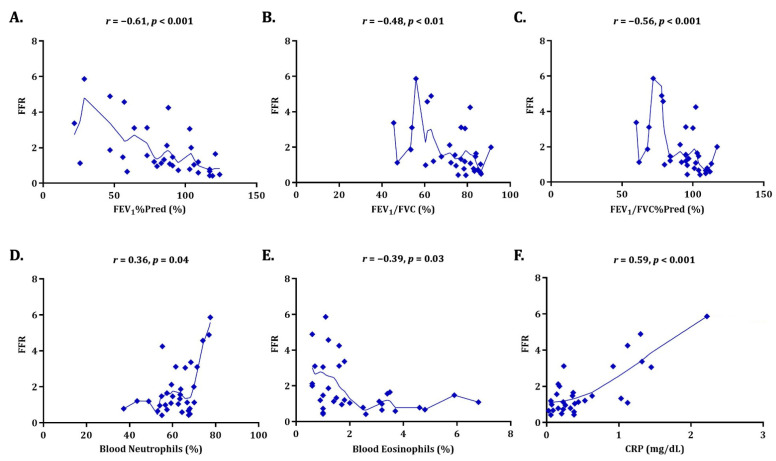
Correlation of FFR with clinical indicators of disease activity and severity. The six correlation plots using the Spearman’s rank correlation coefficient illustrate the relationships of FFR with selected clinical indicators of disease activity and severity, particularly pulmonary function and systemic inflammation. In each plot, the *r* and *p* values are displayed at the top, and the locally estimated scatterplot smoothing (LOESS) line is included. The relationships appeared to be moderate in size (*r* ≈ 0.50–0.60), which is appropriate for preliminary exploratory findings. Panels (**A**–**C**): There was a negative correlation of FFR with pulmonary function, particularly FEV_1_%Pred (*r* = −0.61, *p* < 0.001), FEV_1_/FVC (*r* = −0.48, *p* < 0.01), and FEV_1_/FVC%Pred (*r* = −0.56, *p* < 0.001). Panel (**D**): There was a positive correlation of FFR with percentage of blood neutrophils (*r* = 0.36, *p* = 0.04). Panel (**E**): There was a negative correlation of FFR with percentage of blood eosinophils (*r* = −0.39, *p* = 0.03). Panel (**F**): There was a positive correlation of FFR with serum CRP (*r* = 0.59, *p* < 0.001). CRP: C-reactive protein; FEV_1_: forced expiratory volume in the 1st second; FEV_1_%Pred: percentage of predicted value of FEV_1_; FEV_1_/FVC%Pred: percentage of predicted value of FEV_1_/FVC; FFR: plasma fibrinogen-to-fractional exhaled nitric oxide ratio; FVC: forced vital capacity.

Pulmonary functional impairment, expressed by FEV_1_%Pred, was positively correlated with FeNO (*r* = 0.47, *p* < 0.01, consistent with lesser disease extent), negatively with fibrinogen (*r* = −0.48, *p* < 0.01, consistent with greater systemic inflammation) and, more strongly and significantly, with FFR (*r* = −0.61, *p* < 0.001, Figure 2A). Similar correlations were also observed with FEV_1_/FVC (Figure 2B) and, more significantly, FEV_1_/FVC%Pred (Figure 2C), further showing the clinical importance of the personalised expected FEV_1_/FVC, beyond its absolute measured value. Regarding radiological extent, only fibrinogen was positively correlated with the number of affected lung lobes (*r* = 0.31, *p* = 0.04). The validated severity scores BSI and FACED were both positively correlated with fibrinogen (*r* = 0.44, *p* < 0.01 and *r* = 0.37, *p* = 0.01, respectively), while they tended to positively correlate with FFR as well.

Significant correlations were observed with the routinely measured markers of systemic inflammatory response. Circulating neutrophils, both as the absolute value and the percentage of total leukocytes, were positively correlated with fibrinogen (*r* = 0.41, *p* < 0.01) and FFR (*r* = 0.45, *p* < 0.01, Figure 2D). Circulating eosinophils, only as the percentage of total leukocytes, were positively correlated with FeNO (*r* = 0.42, *p* = 0.01) and negatively correlated with FFR (*r* = −0.39, *p* = 0.03, Figure 2E). Serum CRP, closely aligned with neutrophilic inflammation, was positively correlated with both fibrinogen (*r* = 0.75, *p* < 0.001) and FFR (*r* = 0.59, *p* < 0.001, Figure 2F). As expected, erythrocyte sedimentation rate was positively correlated with fibrinogen (*r* = 0.49, *p* < 0.01), a major determinant of blood viscosity. The results of the analysis are shown in detail in Table 3.

### 3.4. Differences in Clinical Indicators of Disease Activity and Severity Between Patient Groups Dichotomised Using Fibrinogen, FeNO, and FFR

**Figure 3:**
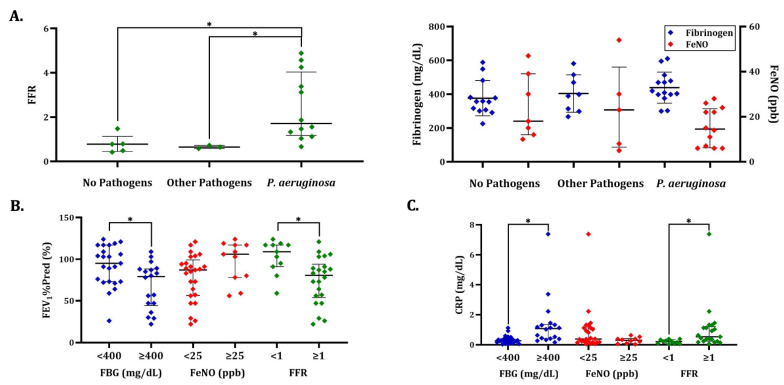
Comparison of fibrinogen, FeNO, and FFR across patients with different isolated airway pathogens and comparison of clinical indicators of disease activity and severity across patient groups dichotomised using fibrinogen, FeNO, and FFR. The simplified box plots created using the Mann–Whitney U test, the Kruskal–Wallis test, the *t*-test, or the one-way analysis of variance, as appropriate, illustrate the differences of fibrinogen, FeNO, and FFR across patients with different isolated airway pathogens as well as the differences of selected clinical indicators of disease activity and severity, particularly pulmonary function and systemic inflammation, across the two patient groups after the dichotomisation of the entire patient population using fibrinogen (<400 mg/dL or ≥400 mg/dL), FeNO (<25 ppb or ≥25 ppb), and FFR (<1 or ≥1). In the simplified box of the normally distributed fibrinogen, the middle line represents the mean, while the upper and lower lines represent the SD. In each simplified box plot of the rest of the non-normally distributed variables, the middle line represents the median, while the upper and lower lines represent the third and first quartiles, respectively. The asterisk (*) indicates the differences with statistical significance (*p* < 0.05). Panel (**A**): Patients with isolation of *P. aeruginosa* in respiratory secretions (with or without isolation of other pathogens) demonstrated significantly higher values of FFR compared to those with isolation of other pathogens only and those with unremarkable respiratory secretion cultures (*p* < 0.01). No significant differences were observed in fibrinogen or FeNO. The three airway pathogen status groups did not significantly differ regarding age or disease extent. Outliers, defined by an absolute modified z-score > 3.5, were excluded from the analysis. Panel (**B**): Patients with fibrinogen ≥ 400 mg/dL and/or FFR ≥ 1 demonstrated significantly lower values of FEV_1_%Pred compared to those with lower values (*p* < 0.01), whereas no significant differences were observed between patients categorised based on FeNO. Panel (**C**): Patients with fibrinogen ≥ 400 mg/dL and/or FFR ≥ 1 demonstrated significantly higher values of serum CRP compared to those with lower values (*p* < 0.001 and *p* < 0.01, respectively), whereas no significant differences were observed between patients categorised based on FeNO. CRP: C-reactive protein; FBG: plasma fibrinogen; FeNO: fractional exhaled nitric oxide; FEV_1_%Pred: percentage of predicted value of forced expiratory volume in the 1st second; FFR: plasma fibrinogen-to-fractional exhaled nitric oxide ratio; *P. aeruginosa*: *Pseudomonas aeruginosa*; SD: standard deviation.

We further dichotomised the entire patient population using fibrinogen, FeNO, and FFR to investigate possibly significant differences in selected clinical indicators of disease activity and severity between the two patient groups. For FFR, we decided to use a cutoff value of 1, as a heuristic, pre-specified rather than an empirically optimised value, corresponding to the widely accepted limits of normality for both fibrinogen (400 mg/dL) and FeNO (25 ppb), which were also used as cutoff values for these two variables. Interestingly, an FFR < 1 was accompanied by worse pulmonary function, as indicated by FEV_1_%Pred (*p* < 0.01), FEV_1_/FVC (*p* = 0.04), and FEV_1_/FVC%Pred (*p* < 0.01), as well as heightened systemic inflammation, as indicated by serum CRP (*p* < 0.01, Figure 3B,C and Table 4). Importantly, only dichotomisation using FFR revealed significant differences in the rate of *P. aeruginosa* isolation, with a higher rate observed in the patients having an FFR ≥ 1 (*p* < 0.01, Table 4).

### 3.5. Discriminative Ability of FFR to Differentiate Bronchiectasis Patients with Favourable and Unfavourable Clinical Indicators of Disease Activity and Severity

**Figure 4:**
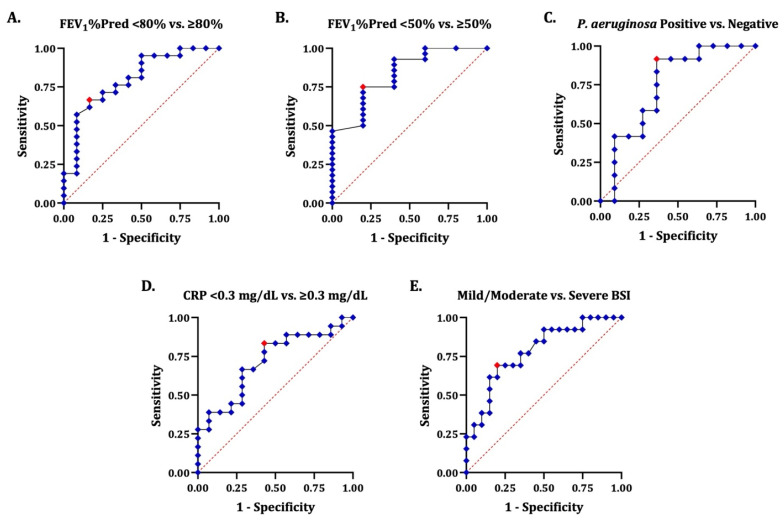
Discriminative ability of FFR to differentiate bronchiectasis patients with favourable and unfavourable clinical indicators of disease activity and severity. The receiver operating characteristic curves illustrate the discriminative ability of FFR to differentiate bronchiectasis patients based on selected clinical indicators of disease activity and severity, particularly pulmonary function, airway infection, systemic inflammation, and validated severity scores. The J point in each curve is depicted in red colour. Panel (**A**): Discriminative ability of FFR for patients with FEV_1_%Pred < 80% vs. ≥80% (AUC = 0.80, 95% CI = 0.64–0.96, J = 0.50, optimal threshold = 1.21). Panel (**B**): Discriminative ability of FFR for patients with FEV_1_%Pred < 50% vs. ≥50% (AUC = 0.83, 95% CI = 0.64–1.00, J = 0.55, optimal threshold = 1.76). Panel (**C**): Discriminative ability of FFR for patients with or without isolation of *P. aeruginosa* in respiratory secretions (AUC = 0.74, 95% CI = 0.53–0.96, J = 0.55, optimal threshold = 0.92). Panel (**D**): Discriminative ability of FFR for patients with serum CRP < 0.3 mg/dL vs. ≥0.3 mg/dL (AUC = 0.72, 95% CI = 0.54–0.90, J = 0.41, optimal threshold = 1.02). Panel (**E**): Discriminative ability of FFR for patients with mild or moderate vs. severe disease according to BSI (AUC = 0.78, 95% CI = 0.62–0.94, J = 0.49, optimal threshold = 1.52). AUC: area under the curve; BSI: Bronchiectasis Severity Index; CI: confidence interval; CRP: C-reactive protein; FEV_1_%Pred: percentage of predicted value of forced expiratory volume in the 1st second; FFR: plasma fibrinogen-to-fractional exhaled nitric oxide ratio; *P. aeruginosa*: *Pseudomonas aeruginosa*; ROC: receiver operating characteristic.

We finally examined the discriminative ability of FFR to differentiate bronchiectasis patients into two pre-defined groups according to selected clinical indicators of disease activity and severity. Interestingly, FFR showed good discriminative ability, with moderate uncertainty, to distinguish the patients with any level of impairment (FEV_1_%Pred < 80%) from those with normal pulmonary function (FEV_1_%Pred ≥ 80%, AUC = 0.80, 95% CI = 0.64–0.96) as well as the patients with severe (FEV_1_%Pred < 50%) from those with moderate, mild, or no impairment (FEV_1_%Pred ≥ 50%, AUC = 0.83, 95% CI = 0.64–1.00, Figure 4A,B). It also showed acceptable, though not outstanding, discriminative ability, with moderate-to-high uncertainty, to distinguish the patients with *P. aeruginosa* isolation (AUC = 0.74, 95% CI = 0.53–0.96, Figure 4C), systemic inflammation indicated by increased levels of serum CRP (AUC = 0.72, 95% CI = 0.54–0.90, Figure 4D), and severe disease according to BSI (AUC = 0.78, 95% CI = 0.62–0.94, Figure 4E).

## 4. Discussion

The biomedical community is in search of novel biomarkers for the personalised endophenotyping of bronchiectasis, which is particularly challenging due to the great heterogeneity of the disease [35]. In this study, we found that FFR was strongly associated with established indices of disease activity and severity. Higher FFR values corresponded to greater pulmonary functional impairment, heightened systemic inflammatory response, and increased rates of *P. aeruginosa* isolation in the lower airway, suggesting that it integrates local and systemic inflammatory signals. These findings point towards the potential utility of FFR as a practical biomarker expressing the balance between neutrophilic, systemic inflammation (indicated by fibrinogen) and eosinophilic, airway inflammation (indicated by FeNO).

**Figure 5:**
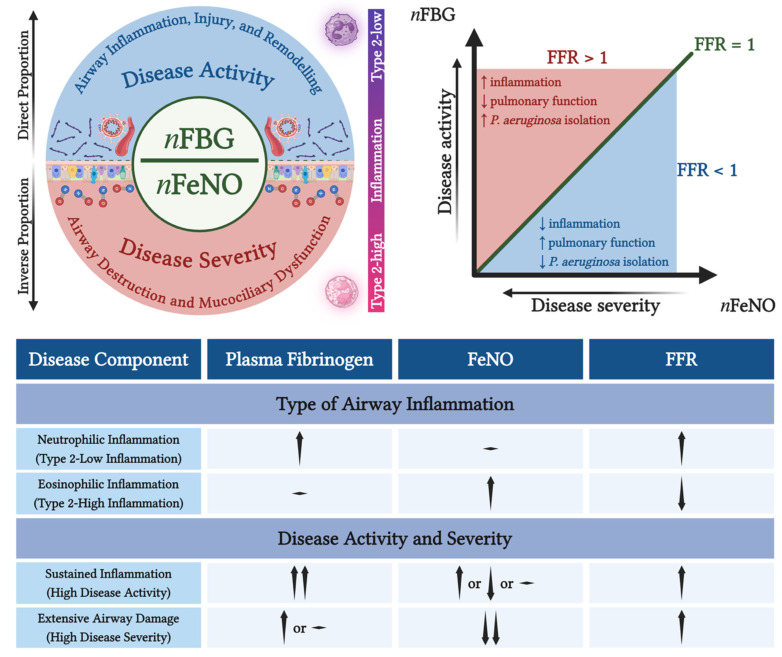
Clinical value of FFR and its fractional components as outlined by their relations to the type of airway inflammation and disease activity and severity. **Left Upper Panel**. FFR derives from the ratio of the normalised values of fibrinogen and FeNO. Fibrinogen is directly proportional to disease activity, i.e., sustained airway inflammation, injury, and remodelling. FeNO is inversely proportional to disease severity, i.e., established airway destruction and mucociliary dysfunction. At the same time, fibrinogen and FeNO are directly proportional to neutrophilic, i.e., type 2-low, and eosinophilic, i.e., type 2-high, airway inflammation. **Right Upper Panel**. An FFR > 1 is associated with heightened systemic inflammation, worse pulmonary function, and higher rate of *P. aeruginosa* isolation. Inversely, an FFR < 1 is associated with dampened systemic inflammation, better pulmonary function, and lower rate of *P. aeruginosa* isolation. **Lower Panel**. Type 2-low airway inflammation is associated with higher fibrinogen levels, resulting in higher FFR. Type 2-high airway inflammation is associated with higher FeNO levels, resulting in lower FFR. Increased disease activity, reflecting sustained inflammation, is associated with higher fibrinogen, accompanied by variable FeNO, depending on disease severity and type of inflammation, thus contributing to higher FFR. Increased disease severity, reflecting extensive airway damage, is associated with lower FeNO, accompanied by higher or normal fibrinogen, depending on disease activity and type of inflammation, thus contributing to higher FFR. *n*FBG: normalised plasma fibrinogen; *n*FeNO: normalised fractional exhaled nitric oxide; FFR: plasma fibrinogen-to-fractional exhaled nitric oxide ratio; *P. aeruginosa*: *Pseudomonas aeruginosa*. Created with: BioRender.com.

The observed association between FFR and the indicators of bronchiectasis activity and severity is plausible in terms of the underlying pathophysiological processes. Interestingly, FFR was associated with markers of neutrophilic or eosinophilic predominance in different directions (Table 3 and Figure 2D,E and Figure 5). On the one hand, fibrinogen, as an acute-phase reactant primarily driven by neutrophil activation, reflects the systemic inflammation that accompanies the chronic infection and tissue injury characterising the extensive airway destruction and remodelling in bronchiectasis [9,10,11,12,15]. Its increased levels in plasma are thus suggestive of high disease activity. On the other hand, FeNO reflects local NO production by the airway epithelium, a process that is typically heightened in type 2-high, eosinophilic inflammation but dampened in bronchiectatic airway epithelial destruction and mucociliary functional impairment [7,21,36]. The decreased levels of FeNO are thus suggestive of high disease severity. As the combination of these two biologically opposite or complementary markers, FFR appears to capture the inflammatory balance and the net activity and severity within the bronchiectatic airway. This dual-domain approach could improve airway disease endotyping, as it emphasises the coexistence and interaction of systemic and local inflammatory processes rather than their separate evaluation (Figure 5).

Previous studies have uniquely linked fibrinogen to a reduction in lung function and to airway infection by *P. aeruginosa*, which are commonly observed in bronchiectasis, as well as with worse validated severity scores and shortened survival [11,12,13,14,15,16]. In concordance with previous observations, our findings point to the clinical importance of fibrinogen in bronchiectasis, as its plasma concentrations were associated with the multifactorial validated severity scores and, more specifically, with pulmonary functional impairment, radiological extent, and systemic inflammation (Table 3 and Figure 1A–C,E). The positive correlation of fibrinogen with circulating neutrophils, but not with eosinophils or FeNO, reflects its link to type 2-low inflammation in bronchiectasis.

The release of NO from the upper and lower airway epithelium is a continuous physiological process that is heightened in response to infection or inflammation [37]. Importantly, FeNO is higher in eosinophilic compared to neutrophilic bronchiectasis [38] and in the presence of favourable clinical features [21,22]. Our findings similarly reveal better pulmonary function and milder disease in patients with higher FeNO (Figure 1E). The levels of FeNO also demonstrated a positive and negative correlation with circulating eosinophils and neutrophils, respectively (Table 3). It is widely speculated that the favourable prognostic value of high FeNO in bronchiectasis is attributed to the predominance of type 2-high inflammation and the resultant greater response to inhaled corticosteroids [21,38]. However, the association of low FeNO with unfavourable clinical features is also explained by the greater extent of NO-deprived, damaged, bronchiectatic airways [21] and the heightened mucociliary clearance impairment in more severe cases, as in cystic fibrosis and primary ciliary dyskinesia [36,39].

FFR was found to more strongly and more significantly associate with pulmonary functional impairment compared to either fibrinogen or FeNO independently, as well as to reliably distinguish patients with any level or a severe level of impairment (Table 3 and Figure 1A, Figure 2A–C and Figure 4A,B). Importantly, only FFR was increased in patients with *P. aeruginosa* isolation and differentiated them from those infected by other pathogens (Table 2 and Figure 1D, Figure 3A and Figure 4C). Patients with severe disease according to BSI presented a higher FFR and were competently distinguished by FFR from those with mild or moderate disease (Figure 1E and Figure 4E). Finally, FFR clearly reflected the magnitude of the systemic inflammatory response and revealed the patients with high inflammation (Table 3 and Figure 2F and Figure 4D). Dichotomising the patient population using FFR led to the differentiation of those with worse pulmonary function, *P. aeruginosa* isolation, and greater systemic inflammation (Table 4 and Figure 5). An FFR > 1 indicates disturbance in at least one of the two components not counterbalanced by a favourable value of the other component. However, as this was a cross-sectional study, the optimal FFR cutoff values that could most accurately distinguish specific bronchiectasis phenotypes remain to be determined through prospective, longitudinal research.

Both fibrinogen and FeNO measurement is widely available in clinical practice and can be efficiently and safely conducted, thus meeting important prerequisites for a potential biomarker [35]. Interestingly, fibrinogen has previously been used as a response biomarker in bronchiectasis in a randomised controlled trial investigating the effect of pulmonary rehabilitation on systemic inflammation [40]. Future studies must focus on the capacity of FFR to stand as a multimodal biomarker in bronchiectasis. Given that FFR showed stronger associations with key clinical features of bronchiectasis compared to either fibrinogen or FeNO independently and may integrate both disease activity and severity by reflecting the interplay between neutrophilic and eosinophilic inflammation, it emerges as a potentially valuable candidate biomarker warranting further validation. In this regard, prospective longitudinal and interventional studies are needed to further clarify its predictive and prognostic value. In concordance with the current therapeutic approach, FFR could possibly reveal patients that benefit from inhaled corticosteroids or other treatment modalities and identify certain treatable traits for the personalised management of bronchiectasis [41].

Our study is the first to concurrently evaluate fibrinogen and FeNO in bronchiectasis. It introduces FFR as a composite index of disease activity and severity and delineates its multidimensional clinical importance, thus paving the way for its further characterisation as a potential biomarker in bronchiectasis. The major limitation of the study is the relatively small sample size and the absence of a validation cohort, which limit multivariate modelling and generalisability, as well as the resultant failure to demonstrate statistical significance at several points as contrasted to previous observations or reasonable evidence-based expectations, especially in the subjectively reported symptoms and patient outcomes. The cross-sectional design also precludes causal inference or longitudinal assessment of changes as well as correlation with prognostic variables, such as exacerbation rate, hospitalisation rate, and mortality. Due to the limited sample size, multivariable adjustment was not possible, so the possibility of residual confounding remains. The study also does not investigate the airway inflammatory constitution or other players in systemic inflammatory response, such as immune cell subpopulations and key inflammatory mediators.

## 5. Conclusions

On the background of the previously demonstrated clinical importance of fibrinogen and FeNO in bronchiectasis, FFR is highlighted as an index associated dually with neutrophilic and eosinophilic inflammation and relating with crucial aspects of disease activity and severity, including pulmonary functional impairment, *P. aeruginosa* isolation, systemic inflammatory response, and validated severity scores. In summary, this study introduces FFR as a novel composite biomarker that integrates systemic and airway inflammatory components in bronchiectasis. Although exploratory, these findings warrant validation in larger, longitudinal cohorts to determine the prognostic value and clinical utility of FFR within personalised management frameworks.

## Figures and Tables

**Table 1:** Summarised descriptive clinical features, laboratory findings, and patient-reported outcomes of the 48 bronchiectasis patients. All data are presented as mean ± SD for normally distributed continuous variables, median (IQR) for non-normally distributed continuous variables, or *N* (%) for categorical variables. Tuberculous causes are included in the infectious causes of bronchiectasis. Dyspnoea mMRC score includes five levels of severity, i.e., from 0 to 4. Sputum Murray score includes three levels of severity, i.e., mucoid (1), mucopurulent (2), and purulent (3). Percentages of patients with certain isolated pathogens in their lower airway are expressed out of the total number of patients with available respiratory secretion cultures. Patients presenting with *P. aeruginosa* isolation might or might not concurrently present isolation of other pathogens, whereas patients presenting isolation of other pathogens did not concurrently present isolation of *P. aeruginosa*. The eight domains of QOL-B quantified, in ascending order, physical functioning (D1), role functioning (D2), vitality (D3), emotional functioning (D4), social functioning (D5), treatment burden (D6), health perceptions (D7), and respiratory symptoms (D8). BEC: blood eosinophil count; BMI: body mass index; BNC: blood neutrophil count; BRICS: Bronchiectasis Radiologically Indexed Computed Tomography Score; BSI: Bronchiectasis Severity Index; COPD: chronic obstructive pulmonary disease; CRP: C-reactive protein; ESR: erythrocyte sedimentation rate; FACED: FEV_1_–Age–Colonisation by *P. aeruginosa*–Radiological Extent–Dyspnoea; FeNO: fractional exhaled nitric oxide; FEV_1_: forced expiratory volume in the 1st second; FFR: plasma fibrinogen-to-fractional exhaled nitric oxide ratio; FVC: forced vital capacity; IgE: immunoglobulin E; IQR: interquartile range; mMRC: modified Medical Research Council; *P. aeruginosa*: *Pseudomonas aeruginosa*; QOL-B-D1–8: domain scores 1–8 of the Quality of Life–Bronchiectasis score; WBC: white blood cells; SD: standard deviation; %Pred: percentage of predicted value.

Variable	Mean ± SD, Median (IQR), or *N* (%)	Variable	Mean ± SD, Median (IQR), or *N* (%)
**Demographic and Somatometric Features**		**Isolated Airway Pathogens**	
Males/Females	16 (33.3%)/32 (66.7%)	*P. aeruginosa*	14 (38.9%)
Age (years)	69.7 (22.3)	Other Pathogens	8 (22.2%)
BMI (kg/m^2^)	25 (5.1)	No Pathogens	14 (38.9%)
**Bronchiectasis Cause**		**Laboratory Findings**	
Idiopathic	21 (43.8%)	WBC (cells/μL)	7.6 (2.6)
Post-infectious	11 (23%)	BNC (cells/μL)	4.7 (2.5)
Asthma	7 (14.6%)	BEC (cells/μL)	0.1 (0.1)
COPD	7 (14.6%)	Neutrophils (%)	62.6 ± 9.9
Chronic aspiration	2 (4.2%)	Eosinophils (%)	1.6 (2.2)
**Respiratory Symptoms**		ESR (mm/1 h)	20 (26)
mMRC Score	1 (0.5)	Serum CRP (mg/dL)	0.4 (0.7)
Murray Score	2 (2)	Serum IgE (IU/mL)	34.6 (61.4)
**Pulmonary Function**		Fibrinogen (mg/dL)	399.9 ± 95
FEV_1_ (L)	2 ± 0.8	FeNO (ppb)	18 (15.5)
FEV_1_%Pred (%)	87.5 (40.5)	FFR	1.2 (1.3)
FVC (L)	2.7 ± 0.8	**Disease Severity**	
FVC%Pred (%)	85.3 ± 24	BSI	7 (7.3)
FEV_1_/FVC (%)	76.4 (17.6)	FACED	3 (3)
FEV_1_/FVC%Pred (%)	94.5 ± 16	**Quality of Life**	
**Radiological Extent**		QOL-B-D1 Score	53.3 (66.7)
Affected Lung Lobes	4 (3)	QOL-B-D2 Score	73.3 (40)
Cylindrical Lesions	35 (72.9%)	QOL-B-D3 Score	63.5 ± 21.4
Varicose Lesions	9 (18.8%)	QOL-B-D4 Score	75 (25)
Cystic Lesions	4 (8.3%)	QOL-B-D5 Score	75 (52.1)
Emphysema	14 (29.2%)	QOL-B-D6 Score	88.9 (44.4)
Tree-in-bud	23 (47.9%)	QOL-B-D7 Score	57.8 ± 23.7
BRICS	1 (1)	QOL-B-D8 Score	77.8 (29.6)

**Table 2:** Differences in fibrinogen, FeNO, FFR, BNC, and BEC across bronchiectasis patient groups stratified by the isolated airway pathogens. Cultures of respiratory secretions, either spontaneous sputum, induced sputum, or bronchoalveolar lavage fluid, were considered acceptable. In the first category, the isolation of *P. aeruginosa* might or might not be accompanied by the isolation of other pathogens. In the second category, only non-*P. aeruginosa* pathogens were isolated. In the third category, no pathogens were isolated. Data are presented as mean ± standard deviation for normally distributed variables and median (interquartile range) for non-normally distributed variables. Differences of the five variables across the three patient groups were investigated using the parametric one-way analysis of variance or the non-parametric Kruskal–Wallis test, as appropriate. Outliers, defined by an absolute modified z-score of more than 3.5 were excluded from the analysis. Statistical significance was set at a *p* of <0.05 and is indicated by an asterisk (*). BEC: blood eosinophil count; BNC: blood neutrophil count; FeNO: fractional exhaled nitric oxide; FFR: plasma fibrinogen-to-FeNO ratio; *P. aeruginosa*: *Pseudomonas aeruginosa*.

Variable	Isolated Airway Pathogens			*p*-Value
	*P. aeruginosa*	Other Pathogens	No Pathogens	
Fibrinogen (mg/dL)	438.3 ± 92.2	403.7 ± 110.9	375.8 ± 104.3	0.281
FeNO (ppb)	14.5 (15.8)	23 (22)	18 (21)	0.367
FFR	1.7 (2.3)	0.7 (0.1)	0.8 (0.3)	0.002 *
BNC (cells/μL)	4.9 (1)	5.6 (5.7)	3.8 (2.3)	0.264
BEC (cells/μL)	0.1 (0.1)	0.1 (0.1)	0.2 (0.1)	0.846

**Table 3:** Correlations of fibrinogen, FeNO, and FFR with clinical indicators of disease activity and severity and patient-reported outcomes. Correlations of plasma fibrinogen, FeNO, and FFR with different clinical variables, including pulmonary function, radiological extent, validated severity scores, inflammatory markers, respiratory symptoms, and patient-reported outcome measure scores, are shown. Correlations were investigated using the parametric Pearson’s correlation coefficient or the non-parametric Spearman’s rank correlation coefficient, as appropriate. Correlations with statistical significance (*p* < 0.05) are illustrated in bold and indicated by an asterisk (*), while trends (0.05 ≤ *p* < 0.10) are indicated by a dagger (^†^). Positive and negative correlations are illustrated in blue and red font, respectively. The eight domains of QOL-B quantified, in ascending order, physical functioning (D1), role functioning (D2), vitality (D3), emotional functioning (D4), social functioning (D5), treatment burden (D6), health perceptions (D7), and respiratory symptoms (D8). BEC: blood eosinophil count; BMI: body mass index; BNC: blood neutrophil count; BRICS: Bronchiectasis Radiologically Indexed Computed Tomography Score; BSI: Bronchiectasis Severity Index; CRP: C-reactive protein; ESR: erythrocyte sedimentation rate; FACED: FEV_1_–Age–Colonisation by *Pseudomonas aeruginosa*–Radiological Extent–Dyspnoea; FeNO: fractional exhaled nitric oxide; FEV_1_: forced expiratory volume in the 1st second; FFR: plasma fibrinogen-to-FeNO ratio; FVC: forced vital capacity; mMRC: modified Medical Research Council; QOL-B-D1–8: domain scores 1–8 of the Quality of Life–Bronchiectasis score; WBC: white blood cells; %Pred: percentage of predicted value.

Variable	Spearman’s *rho* or Pearson’s *r* (*p*-Value)		
	Fibrinogen (*n* = 45)	FeNO (*n* = 36)	FFR (*n* = 33)
**Age and BMI**			
Age (years)	−0.015 (0.922)	0.137 (0.426)	−0.247 (0.167)
BMI (kg/m^2^)	−0.040 (0.837)	−0.016 (0.941)	−0.029 (0.902)
**Pulmonary Function**			
FEV_1_%Pred (%)	−0.476 (0.002) *	0.470 (0.004) *	−0.606 (<0.001) *
FVC%Pred (%)	−0.468 (0.002) *	0.360 (0.031) *	−0.451 (0.009) *
FEV_1_/FVC (%)	−0.420 (0.006) *	0.312 (0.064) ^†^	−0.483 (0.004) *
FEV_1_/FVC%Pred (%)	−0.384 (0.013) *	0.373 (0.025) *	−0.561 (<0.001) *
**Radiological Extent**			
Affected Lung Lobes	0.308 (0.042) *	−0.177 (0.301)	0.163 (0.372)
BRICS	0.204 (0.185)	−0.064 (0.714)	0.118 (0.513)
**Validated Severity Scores**			
BSI	0.440 (0.003) *	−0.268 (0.114)	0.299 (0.091) ^†^
FACED	0.372 (0.012) *	−0.316 (0.061) ^†^	0.327 (0.063) ^†^
**Laboratory Findings**			
Blood WBC (cells/μL)	0.373 (0.012) *	−0.411 (0.013) *	0.471 (0.006) *
BNC (cells/μL)	0.409 (0.005) *	−0.404 (0.014) *	0.452 (0.008) *
BEC (cells/μL)	0.059 (0.703)	0.311 (0.065) ^†^	−0.246 (0.168)
Blood Neutrophils %	0.425 (0.004) *	−0.301 (0.074) ^†^	0.357 (0.042) *
Blood Eosinophils %	−0.067 (0.661)	0.416 (0.012) *	−0.390 (0.025) *
ESR (mm/1 h)	0.493 (0.001) *	0.241 (0.163)	0.023 (0.897)
Serum CRP (mg/dL)	**0.750 (<0.001)**	−0.330 (0.057) ^†^	0.592 (<0.001) *
Serum IgE (IU/mL)	0.045 (0.788)	0.245 (0.156)	−0.098 (0.588)
Fibrinogen (mg/dL)	–	−0.163 (0.366)	–
FeNO (ppb)	−0.163 (0.366)	–	–
**Respiratory Symptoms and Patient-Reported Outcomes**			
mMRC Score	0.258 (0.087) ^†^	−0.275 (0.105)	0.283 (0.110)
Murray Score	−0.021 (0.903)	0.137 (0.479)	−0.054 (0.773)
QOL-B-D1 Score	−0.175 (0.263)	0.192 (0.269)	−0.178 (0.330)
QOL-B-D2 Score	−0.130 (0.406)	0.138 (0.430)	−0.132 (0.471)
QOL-B-D3 Score	−0.041 (0.793)	0.133 (0.448)	−0.019 (0.916)
QOL-B-D4 Score	−0.257 (0.097) ^†^	0.019 (0.913)	−0.074 (0.686)
QOL-B-D5 Score	−0.171 (0.273)	−0.072 (0.683)	0.054 (0.728)
QOL-B-D6 Score	0.065 (0.754)	0.240 (0.296)	−0.083 (0.745)
QOL-B-D7 Score	−0.156 (0.320)	−0.071 (0.685)	0.031 (0.865)
QOL-B-D8 Score	−0.130 (0.405)	0.015 (0.932)	−0.067 (0.714)

**Table 4:** Differences in clinical indicators of disease activity and severity between patient groups dichotomised using fibrinogen, FeNO, and FFR. Differences in selected clinical variables, based on the findings of the exploratory data analysis, including pulmonary function, inflammatory markers, and disease severity scores, are shown between patient groups dichotomised by plasma fibrinogen, FeNO, and FFR. Data are presented as mean ± SD for normally distributed continuous variables, median (IQR) for non-normally distributed continuous variables, and percentages for categorical variables. Differences of the continuous variables across the two patient groups were investigated using the parametric *t*-test or the non-parametric Mann–Whitney U test, as appropriate, while the differential presence of isolated *P. aeruginosa* was investigated using Fischer’s exact test. Differences with statistical significance (*p* < 0.05) are illustrated in bold and indicated by an asterisk (*). BEC: blood eosinophil count; BNC: blood neutrophil count; BSI: Bronchiectasis Severity Index; CRP: C-reactive protein; FACED: FEV_1_–Age–Colonisation by *P. aeruginosa*–Radiological Extent–Dyspnoea; FeNO: fractional exhaled nitric oxide; FEV_1_: forced expiratory volume in the 1st second; FFR: plasma fibrinogen-to-FeNO ratio; FVC: forced vital capacity; *P. aeruginosa*: *Pseudomonas aeruginosa*; WBC: white blood cells; %Pred: percentage of predicted value.

Variables	Plasma Fibrinogen (mg/dL)			FeNO (ppb)			FFR		
	<400	≥400	*p*-Value	<25	≥25	*p*-Value	<1	≥1	*p*-Value
FEV_1_%Pred (%)	95 (40)	79 (40.8)	0.007 *	87 (38)	106 (38)	0.077	109 (24)	80.5 (34.3)	0.003 *
FVC%Pred (%)	93.2 ± 22.4	74.8 ± 21.6	0.015 *	83 ± 23.8	95.8 ± 20.9	0.241	111 (29)	86.5 (24.5)	0.079
FEV_1_/FVC (%)	81.4 (8.1)	67.8 (17.3)	0.009 *	77 (20.2)	79.3 (9.1)	0.327	82.9 (8)	73.3 (19.1)	0.036 *
FEV_1_/FVC%Pred (%)	98.7 ± 14	88 ± 17.1	0.100	91.8 ± 16.7	100.2 ± 10.2	0.558	105 (11)	93.5 (23.3)	0.242
BSI	6 (5)	11 (7.5)	0.009 *	7 (6)	6 (3.5)	0.291	6 (2.5)	9 (8.5)	0.251
FACED	2 (2)	3 (1.5)	0.033 *	3 (3)	2 (1.5)	0.064	2 (1.5)	3 (3)	0.270
Blood WBC (cells/μL)	7.3 (2.4)	8 (4.8)	0.065	7.9 (2.5)	7.2 (1.4)	0.248	7.2 (1.8)	7.6 (2.5)	0.294
BNC (cells/μL)	4.4 (2)	5.2 (3.4)	0.080	4.7 (1.9)	4.1 (1.9)	0.231	4 (1.5)	4.7 (1.9)	0.214
BEC (cells/μL)	0.1 (0.2)	0.1 (0.1)	0.972	0.1 (0.1)	0.2 (0.1)	0.266	0.2 (0.1)	0.1 (0.1)	0.251
Blood Neutrophils %	60.5 ± 10.1	64.2 ± 8.8	0.503	63.4 (12.5)	63.2 (12.9)	0.331	57.4 (12.9)	63.5 (10.2)	0.240
Blood Eosinophils %	1.7 (2.4)	1.6 (1.4)	0.606	1.4 (2.1)	1.9 (1.6)	0.208	2.6 (2.1)	1.5 (1)	0.120
Serum CRP (mg/dL)	0.3 (0.2)	1.1 (0.9)	<0.001 *	0.4 (0.9)	0.3 (0.3)	0.115	37.9 (83.6)	29.1 (42)	0.002 *
Fibrinogen (mg/dL)	–	–	–	398.2 (96.9)	374.8 (156.6)	0.916	–	–	–
FeNO (ppb)	21 (14)	13 (16.5)	0.132	–	–	–	–	–	–
FFR	–	–	–	–	–	–	–	–	–
*P. aeruginosa* Isolation (%)	25%	60%	0.080	58.8	28.6	0.371	12.5	73.3	0.009 *

## Data Availability

Data supporting the results of this study are available from the corresponding author upon reasonable request.

## Abbreviations

The following abbreviations are used in this manuscript:

AUC	Area under the curve
BEC	Blood eosinophil count
BNC	Blood neutrophil count
BMI	Body mass index
BRICS	Bronchiectasis Radiologically Indexed Computed Tomography Score
BSI	Bronchiectasis Severity Index
CI	Confidence interval
COPD	Chronic obstructive pulmonary disease
CRP	C-reactive protein
ESR	Erythrocyte sedimentation rate
FACED	FEV_1_–Age–Colonisation by *P. aeruginosa*–Radiological Extent–Dyspnoea
FeNO	Fractional exhaled nitric oxide
FEV_1_	Forced expiratory volume in the 1st second
FFR	Plasma fibrinogen-to-fractional exhaled nitric oxide ratio
FVC	Forced vital capacity
IgE	Immunoglobulin E
IQR	Interquartile range
mMRC	Modified Medical Research Council
NO	Nitric oxide
%Pred	Percentage of predicted value
*P. aeruginosa*	*Pseudomonas aeruginosa*
ROC	Receiver operating characteristic (curve)
SD	Standard deviation
QOL-B	Quality of Life–Bronchiectasis
WBC	White blood cells
